# From “cutaneous anthrax” to “primary delusional infestation”

**DOI:** 10.1192/j.eurpsy.2023.948

**Published:** 2023-07-19

**Authors:** I. A. Ferreira, A. Ponte

**Affiliations:** Psychiatry, Hospital do Divino Espírito Santo, Ponta Delgada, Portugal

## Abstract

**Introduction:**

Primary delusional infestation is a rare psychiatric condition in which patients mistakenly believe that their skin or other body parts are infested by small, living organisms, despite the fact that no organisms can be found upon investigation. The delusion occurs concurrently with abnormal cutaneous sensations. Therefore, they typically have a history of prior negative evaluations by dermatologists and general practice physicians. In addition, patients may have also received repeated courses of dermatologic and anti-infective therapies, despite the lack of an objective diagnosis.

**Objectives:**

To describe the clinical case of a patient who suffered from an undiagnosed primary delusional infestation for 12 years.

**Methods:**

Description of a clinical case and a non-systematic review of the literature.

**Results:**

We describe the clinical case of a 65-year-old woman who spent 12 years being evaluated by multiple medical and surgical specialties for the following complaint: “sensation of something moving beneath the skin.” At the onset of the complaint, the patient believed that this “strange sensation” was due to a recent tooth procedure. However, as she felt the discomfort not improving, she believed it to be a consequence of a cutaneous *anthrax* infection. Thus, the patient started using tweezers to grasp the living organism that was bothering her and that behavior caused intense scaring in her face. Meanwhile, the patient was also submitted to 3 cutaneous biopsies (prescribed by a dermatologist) that refuted the hypothesis of any living organism underneath her skin. In addition, the patient was prescribed sertraline, bromazepam and lorazepam that, although improved her sleep and anxiety levels, were inefficient to treat the root of her suffering. Finally, after 12 years of dispersed medical follow-up, this patient was evaluated by a new psychiatrist and prescribed paliperidone that rapidly made the agonizing “strange sensation” disappear.

**Conclusions:**

Even though primary delusional infestations are a rare psychiatric diagnosis, all medical doctors should consider it when their patients report bothersome dermatologic complaints associated with the belief of infestation and negative diagnostic examinations. It is incredibly important to consider this diagnostic, as the early treatment of this entity might prevent the patient from undergoing multiple years of suffering and discomfort.

**Disclosure of Interest:**

None Declared

